# A therapeutic exploratory study to determine the efficacy and safety of calcineurin-inhibitor-free de-novo immunosuppression after liver transplantation: CILT

**DOI:** 10.1186/1471-2482-10-15

**Published:** 2010-04-09

**Authors:** Armin D Goralczyk, Andreas Schnitzbauer, Tung Y Tsui, Giuliano Ramadori, Thomas Lorf, Aiman Obed

**Affiliations:** 1 Department of General and Visceral Surgery, University Medical Center Göttingen, Göttingen, Germany; 2 Klinik und Poliklinik für Chirurgie, Universitätsklinikum Regensburg, Regensburg, Germany; 3 Department of Hepatobiliary and Transplant Surgery, Universitätsklinikum Hamburg-Eppendorf, Hamburg, Germany; 4 Department of Gastroenterology and Endocrinology, University Medical Center Göttingen, Göttingen, Germany

## Abstract

**Background:**

Immunosuppression with calcineurin inhibitors (CNI) increases the risk of renal dysfunction after orthotopic liver transplantation (OLT). Controlled trials have shown improvement of renal function in patients that received delayed and/or reduced-dose CNI after OLT. Delaying immunosuppression with CNI in combination with induction therapy does not increase the risk of acute rejection but reduces the incidence of acute renal dysfunction. Based on this clinical data this study protocol was designed to assess the efficacy and safety of calcineurin-inhibitor-free *de-novo *immunosuppression after liver transplantation.

**Methods/Design:**

A prospective therapeutic exploratory, non-placebo controlled, two stage monocenter trial in a total of 29 liver transplant patients was designed to assess the safety and efficacy of *de-novo *CNI-free immunosuppression with basiliximab, mycophenolate sodium, prednisolone and everolimus. The primary endpoint is the rate of steroid resistant rejections. Secondary endpoints are the incidence of acute rejection, kidney function (assessed by incidence and duration of renal replacement therapy, incidence of chronic renal failure, and measurement glomerular filtration rate), liver allograft function (assessed by measurement of AST, ALT, total bilirubin, AP, GGT), treatment failure, (i. e., re-introduction of CNI), incidence of adverse events, and mortality up to one year after OLT.

**Discussion:**

This prospective, two-stage, single-group pilot study represents an intermediate element of the research chain. If the data of the phase II study corroborates safety of *de-novo *CNI-free immunosuppressive regimen this should be confirmed in a randomized, prospective, controlled double-blinded clinical trial. The exploratory data from this trial may then also facilitate the design (e. g. sample size calculation) of this phase III trial.

**Trial registration number:**

NCT00890253 (clinicaltrials.gov)

## Background

Recipients of a liver allograft are at high risk of acute and subsequently chronic renal dysfunction resulting in a significantly increased risk of premature death [1,2]. After OLT more than 90% of patients receive an immunosuppressive regimen based on calcineurin inhibitors (CNI), i. e., cyclosporine A(CsA) or tacrolimus (TAC) [3]. CNI cause renal arteriolopathy resulting in histopathological and functional changes [4]. Hence, nephrotoxicity associated with CNI mitigates renal function and contributes to the increased risk of end-stage renal disease after OLT [5-7]. Strategies are needed to minimize the incidence of renal impairment after OLT.

### Pathophysiology of CNI-induced nephropathy

Despite major differences in the chemical structure, both, TAC and CsA, seem to cause nephropathy characterized by vasoconstriction of renal arterioles [4]. The clinical manifestations of this acute renal dysfunction include reduction in glomerular filtration rate (GFR), hypertension, hyperkalemia, tubular acidosis, increased reabsorption of sodium and oliguria [8]. This acute form of CNI toxicity may be reversed when CNI administration is reduced or withdrawn. In contrast, the chronic form of CNI-induced nephrotoxicity is characterized not only by renal vasoconstriction but also by the development of structural damage, including arteriolopathy and tubulointerstitial fibrosis, which is irreversible and may lead to end-stage renal disease [4].

### Immunosuppressive regimens to avoid CNI

Two main strategies to avoid the detrimental effects of CNI on kidney function have been evaluated in clinical trials. Long-term kidney damage may be attenuated by reduction or even withdrawal of CNI some months after OLT while maintaining adequate immunosuppression by adding inosine monophosphate dehydrogenase (IMPDH) inhibitors or mammalian target-of-rapamycin (mTOR) inhibitors [9-13]. It has been shown that regime change does not result in higher rejection rate but improves kidney function.

However, not all patients seem to profit from this strategy, possibly because irreversible kidney damage has already taken place.

Alternatively, it has been shown in other studies that administration of CNI may be delayed until the fifth post-operative day (POD) or even later [14-17]. Adequate immunosuppression in the early phase ofter OLT was maintained with the perioperative administration of interleukin 2-receptor (IL2R) antibodies (Ab) or antithymocyte globuline (ATG). Neuberger et al. conducted a randomized, prospective, open-label trial in patients with good pretransplant kidney function in which reduced dose tacrolimus (trough levels ≤ 8 ng/mL) was delayed until the fifth day post transplant in combination with mycophenolate mofetil, corticosteroids and induction with daclizumab; the primary endpoint was change from baseline in estimated glomerular filtration rate (eGFR) at 52 weeks [14]. They conclude that this regimen was associated with less nephrotoxicity compared to therapy with standard-dose tacrolimus and corticosteroids without compromising efficacy or tolerability. The beneficial effect of delaying CNI also been shown before in smaller trials with a different study population or induction therapy [15-17].

Thus avoiding acute CNI-associated kidney damage ameliorates long-term kidney function even in patients with good pretransplant kidney function. This effect should even be more pronounced in patients with compromised pretransplant kidney function, a subgroup of patients coming more and more into focus in the MELD era.

### Safety and efficacy of the investigational medicinal products

Basiliximab, everolimus, and enteric-coated mycophenolate sodium (EC-MPS) are the investigational medicinal products that will be evaluated in this clinical trial.

Basiliximab has been shown to reduce the incidence of acute rejection with no clinically relevant safety or tolerability concerns [18,19]. Both IL2R antibodies, i. e., basiliximab and daclizumab, have also been shown to prevent acute rejection even when CNIs are delayed for five or more days after OLT [14,17]. Since daclizumab has been withdrawn from the market we use basiliximab as standard immunoprophylaxis in our transplant center and in this study.

For maintenance immunosuppression the proposed regimen will include the mTOR inhibitor everolimus. Phase I and II trials of therapy with everolimus in *de novo *liver transplant recipients implicate efficacy and good tolerability of this immunosuppressant [20,21]. We decided to use everolimus and not sirolimus because i) dose adjustments are more convenient with the shorter biological half-life of everolimus (28 hrs) compared to sirolimus (61-72 hrs); ii) increased rates of hepatic artery thrombosis and wound healing disturbances have been observed in a trial involving sirolimus but not in trials involving everolimus [20,21].

Nonetheless everolimus therapy will be delayed until the 10th postoperative day for safety reasons. Concomitantly the antimetabolite EC-MPS will be used instead of the commonly used mycophenolate mofetil (MMF). MMF may cause considerable gastro-intestinal adverse reactions, e. g. diarrhea, and reduction or discontinuation is not uncommon even in the immediate posttransplant period. EC-MPS has been shown to cause less adverse reactions und thus compliance with the study protocol may be better.

EC-MPS will also be administered in a higher than usual dose, i. e., 2160 mg/day instead of 1440 mg/day in two doses, because van Gelder et al. suggested that higher MPAexp osures in the early post-transplantation period would be required in patients on reduced rather than standard cyclosporine doses [22].

### Study rationale

Generally, avoidance of CNIs improves kidney function and does not result in higher rate of rejection when an adequate level of immunosuppression is maintained, e. g. by use of non-nephrotoxic immunosuppressive agents such as mTOR-inhibitors and mycophenolate, and/or concomitant interleukin-2 receptor blockade by induction with anti-CD25 antibodies [9-17,23,24]. However, non of the mentioned studies investigated the effect of a CNI-free *de-novo *regimen in patients that underwent liver transplantation.

Based on the aforementioned clinical data this study protocol is designed to show primarily the safety of CNI-free *de-novo *immunosuppression with basiliximab, everolimus, EC-MPS, and corticosteroids in patients after OLT with impaired pretransplant kidney function. To determine whether this regimen has sufficient safety and efficacy to warrant more extensive development and to obtain preliminary data for sample size estimation and planning of a phase III clinical trial this phase II pilot study is designed as a prospective, non-randomized, open-label study. If the CNI-free *de-novo *immunosuppressive regimen gives adequate immunosuppressive protection and is safe with regards to toxicity, a phase III clinical trial will be conducted.

## Methods & Design

Research was carried out in compliance with the Helsinki Declaration. After completion of trial design and approval by the competent authorities in Germany and the European Union an ethics vote was obtained from the ethics committee at the University Medical Center, Göttingen, Germany before start of enrollment. Only patients who meet the inclusion and exclusion criteria (see Table 1) are considered for enrollment. After enrollment patients will be followed-up for one year. The schedule for all study related activities and data collection is listed in Table 2.

**Table 1:** Criteria for inclusion and exclusion of patients.

Inclusion criteria	Exclusion criteria
1. Patients undergoing primary liver transplantation.	1. Pregnant or nursing women.
2. Patients older than 18 years.	2. Patients with a psychological, familial, sociologic or geographic condition potentially hampering compliance with the study protocol and follow-up schedule.
3. Patients with a hepatorenal syndrome.	3. Patients under guardianship, i. e., individuals who are not able to freely give their informed consent.
4. Female patients of childbearing potential willing to perform a highly effective contraception during the study and 12 weeks after conclusion of study participation. ^†^	4. Patients with pre-transplant renal replacement therapy > 14 days.
5. eGFR < 50 ml/min at the time point of transplantation.	5. Patients with a reason for renal impairment other than a hepatorenal syndrome.
6. Serum creatinine levels > 1.5 mg/dL at the time-point of transplantation.	6. Patients with a known hypersensitivity to any of the investigational medicinal products.
	7. Patients with thrombocytopenia (<50.000/nl), hypertriglyceridemia (> 350 mg/dl), or hypercholesterinemia (> 300 mg/dl), and patients with signs of hepatic artery stenosis prior to initiation of therapy with everolimus.

^† ^A highly effective method of birth control is defined as those which result in a low failure rate (i.e. less than 1 per year) when used consistently and correctly such as implants, injectables, combined oral contraceptives, hormonal intrauterine devices (IUDs), sexual abstinence or vasectomised partners in accordance to CPMP/ICH/286/95.

**Table 2:** Schedule of study related activities and data collection.

Study activity	Day										Month		
		
	-3-0	1	2	3	5	10	14/15	20/21	25/26	30	3	6	12
Patient baseline characteristics and transplant data													

Informed consent	*												
Medical history	*												
Physical examin	*												
Graft history	*												
Pregnancy test	*												

Safety parameters													

Vital signs	*	*	*	*	*	*	*	*	*	*	*	*	*
Blood chemistry^†^	*	*	*	*	*	*	*	*	*	*	*	*	*
Heamogram	*	*	*	*	*	*	*	*	*	*	*	*	*
Coagulation lab^†^	*	*	*	*	*	*	*	*	*	*	*	*	*
MPAtrough level		*	*	*	*	*	*	*	*	*	*	*	*
EVL trough level							*	*	*	*	*	*	*
Ultrasound^‡^		*	*	*		*							
Adverse events		*	*	*	*	*	*	*	*	*	*	*	*
Rejection^¶^		*	*	*	*	*	*	*	*	*	*	*	*

Efficacy parameters													

eGFR	*	*	*	*	*	*	*	*	*	*	*	*	*
Rejection^||^		*	*	*	*	*	*	*	*	*	*	*	*

The chart indicates mandatory measurements and/or recordings by an asterisk (green background color). Blood samples already taken in the routine process at the planned time do not have to be taken twice.

^† ^Mandatory laboratory measurements include: sodium, potassium, creatinine, urea, alanine transaminase, aspartate transaminase, alkaline phosphatase, *γ*-glutamyl transferase, total bilirubin, lactate dehydrogenase, cholesterole, triglycerides, activated partial thromboplastine time and Quick's value.

^‡ ^Additional examinations in case of pathological findings or indication.

^¶ ^If laboratory measurements and/or clinical data indicate rejection the attending transplant surgeon may decide to begin treatment with steroids. If the rejection is clinically resistant to therapy with steroids a liver biopsy has to be obtained. Only if there is histological evidence for steroid resistant rejection it will be classified as such.

^|| ^Rejection is a safety *and *efficacy parameter.

### Treatment

**Figure 1:**
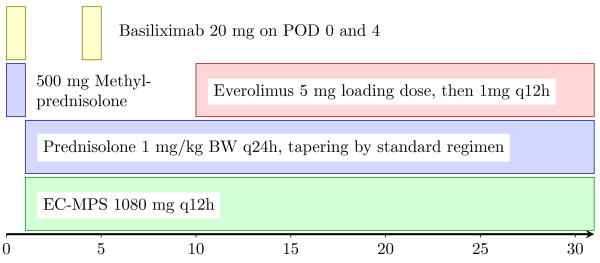
**Scheme of proposed reno-protective immunosuppressive regimen without CNI**. Abbr.: EC-MPS, enteric-coated mycophenolate sodium; POD, post-operative day; BW, body weight; q12h and q24h, every 12 or 24 hours.

Immunosuppressive treatment (see Figure 1) will be initiated at reperfusion of the graft starting with 500 mg methylprednisolone and 20 mg basiliximab given intravenously. The latter will be repeated on the 4th post-operative day (POD).

Maintenance immunosuppression includes EC-MPS, everolimus, and prednisolone. Since systemic exposure of MPAmay be reduced in patients receiving immunosuppressive regimens with reduced dose CNI or without CNI [22] EC-MPS will be given at an increased dose, i. e., 1080 mg q12, starting within 24 hours after OLT.

Earliest, on day 10 after OLT everolimus will be introduced with a loading dose of 5 mg/d. Thereafter a dosage of 2 mg/d in two doses will be administered, aiming at 24 hours trough levels for everolimus between 4 and 8 ng/mL. Steroids (non investigational medicinal product) will be started on day 1 after transplantation with 1 mg/kg body weight and will be tapered every 2 days for 5 mg to a dosage of 20 mg and for 2.5 mg every two days to 7.5 mg. Thereafter the dosage will be reduced to 5 mg and 2.5 mg for 1 week each and eliminated thereafter.

Additionally, every patient will receive Cytomegalo virus (CMV) prophylaxis and prophylaxis against Pneumocystis carinii infection during the first 3 months after liver transplantation.

### Objectives and endpoints

The primary objective of this pilot study is to evaluate the safety and to investigate the preliminary efficacy of a CNI-free immunosuppressive *de-novo *regimen in patients with impaired renal function undergoing liver transplantation. The primary endpoint was thus defined as the incidence of biopsy proven steroid resistant acute rejection(s) within the first 30 days.

Secondary objectives of the trial are the assessment of adverse reactions and renal function under the proposed study regimen. Secondary endpoints will be evaluated after in one interim analysis and after the end of the follow-up period, i. e., one year, and include mortality, the incidence of infection, treatment failure (defined as re-introduction of CNI), wound-healing disturbances, hepatic artery stenoses, and hemato-lymphatic side effects. Furthermore kidney damage will be assessed by the incidence and length of renal replacement therapy and the estimation of renal function using the Modification of Diet in Renal Disease (MDRD) formula. Finally liver allograft function will assessed by measurement of liver enzymes, bile excretion, and coagulation assays.

All end-points will be compared to reference values of CNI based immunosuppression obtained from a meta-analysis [25].

### Sample size calculation

**Figure 2:**
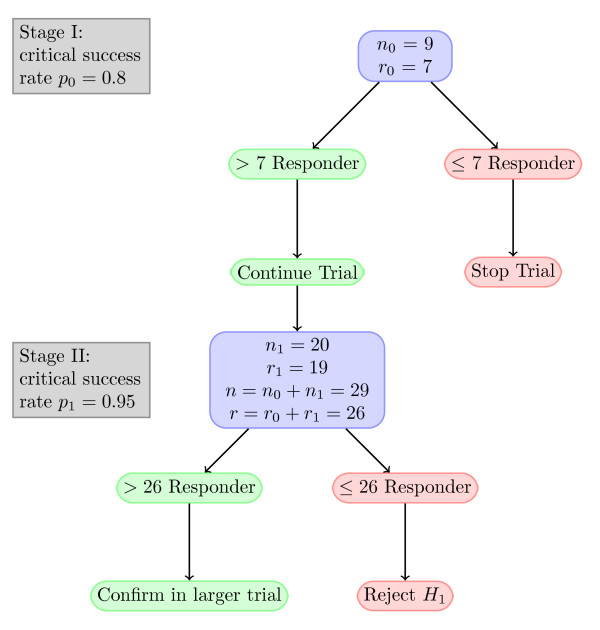
**Flow chart of the CILT study**. Derived from an optimal two-stage design for a phase II clinical trial with the following parameters: *p*_0 _= 0.8, *p*_1 _= 0.95, error probability *α *= 0.05, and error probability *β *= 0.2. Abbr.: *n*, number of patients; *r*, maximum number of responders at which *H*_0 _will not be rejected; *H*_1_, alternative hypothesis.

The sample size calculation is based on the rate of steroid resistant rejection after OLT. According to the pooled estimate of a Cochrane meta-analysis the incidence of steroid resistant rejection in patients with a CNI based immunosuppressive regimen may be inferred as 12.61% with a 95%-confidence interval of [6.36, 18.65] (estimated by bootstrap methods) [25]. To minimize the sample size for this therapeutic exploratory trial the study is designed as a non-controlled, prospective, two-stage study. In this two stage design [26] superiority of the safety or efficacy rate to the required minimum or "uninteresting" rate *p*_0 _will be tested in stage I. If the treatment is inferior to *p*_0 _the trial will be halted and no further patients enrolled, whereas if the treatment is superior to *p*_0 _stage II will be entered. In stage II the rate will be compared to the actual target rate *p*_1_. For the present trial *p*_0 _and *p*_1 _have been fixed at 0.8 and 0.95 respectively. With error probability *α *= 0.05 and power *β *= 0.2 the required sample sizes for stage I and II are *n*_0 _= 9 and *n*_1 _= 20 respectively. The maximum success rates that corroborate inferiority of the regimen to the fixed rates *p*_0 _and *p*_1 _have been calculated as *r*_0 _= 7 and *r*_0 _+ *r*_1 _= 26, meaning that if more than 7 patients in stage I and 26 patients in stage II do not experience a steroid resistant rejection the true response is at least the target level *p*_1 _(see Figure 2).

### Adverse events

All adverse events (AE) are recorded. Events related to the initial diagnosis for liver transplantation, to the transplantation procedure itself, or problems associated with routine procedures after transplantation, i.e. liver biopsy, are not to be noted as AE or serious adverse event (SAE) unless the investigator deems the events to be a cause of the study drug. All SAE potentially associated with the application of study medication must be documented and the sponsor has to be informed by the principal investigator within 24 hours. The sponsor will notify all concerned investigators, the Ethics Committee, and competent authority of findings that could adversely affect the health of subjects. AE will be analysed in an interim analysis and at termination of the trial.

### Quality assurance

The study is performed according to the principles of the ICH-GCP guidelines and the ethical principles according to the current revision of the Declaration of Helsinki and local legal and regulatory requirements.

The trial is monitored by a contract research organization according to standard operation procedures (SOP) that are based on ICH-GCP guidelines. An independent safety board monitors closely the proper conduct of the trial and all SAE reports to ensure the safety of the subjects during the course of the study.

### Statistical analysis plan

The confirmatory analysis will be performed for the per-protocol population. All statistical analyses will be performed with a type I error *α *= 0.05 and, where appropriate, a non-inferiority margin of *δ *= -0.1 will be assumed.

All rates will be tested in an approximate one sample design test for non-inferiority [27] against reference values obtained from a pooled meta-analysis of trials with immunosuppressive regimen based on CNI [25].

Confidence intervals will be calculated by the method of Agresti and Coull [28]. The only confirmatory analysis will concern the primary endpoint; all other analyses will be exploratory.

Continuous secondary end-points will be compared to reference values with student's T test unless there is considerable evidence for a non-parametric distribution. Event-time data, i. e., time to acute rejection, patient, and graft survival will be estimated by the Kaplan-Meier method.

## Discussion

Long term kidney damage after OLT in patients with pre-operative renal dysfunction is a growing problem in the MELD era. CNI, which are the basis of most immunosuppressive regimens, may aggravate the damage and should thus be avoided. Reducing or delaying CNI is safe and reduces renal impairment. But it is not known if complete abdication of CNI may increase the risk of rejection.

Before conducting a controlled, randomized, double-blind phase III study to show efficacy of the drug regimen safety has to be ensured. Also effect size estimates have to be obtained for for planing such a study, e. g. for sample size calculation.

Hence, we designed this pilot study as a two-stage, single-group, uncontrolled trial to assess the incidence of steroid resistant rejection under the proposed treatment. If the phase II study corroborates safety of *de-novo *CNI-free immunosuppression the exploratory data may serve as a fundament for planing and conducting a randomized, prospective, controlled double-blinded clinical trial to confirm the results.

## Competing interests

ADG, TL, and AO have received reimbursements (restricted travel grant) from Novartis Pharma GmbH, Germany. All other authors declare that they have no competing interests.

## Authors' contributions

ADG designed of the study, developed the statistical part of the study protocol and the statistical analysis plan, and developed essential study documents. AS and TYT participated in the design of the study. TL and GR supported the design of the study with their knowledge and experience. AO conceived and designed the study based on his own clinical results. Further he conducts the study as the primary investigator. All authors read and approved the final manuscript

## Pre-publication history

The pre-publication history for this paper can be accessed here:

http://www.biomedcentral.com/1471-2482/10/15/prepub
